# Novel Microsynthesis of High-Yield Gold Nanoparticles to Accelerate Research in Biosensing and Other Bioapplications

**DOI:** 10.3390/bios13120992

**Published:** 2023-11-21

**Authors:** Víctor Díaz-García, Astrid Haensgen, Ligia Inostroza, Braulio Contreras-Trigo, Patricio Oyarzun

**Affiliations:** Facultad de Ingeniería, Arquitectura y Diseño, Universidad San Sebastián, Lientur 1457, Concepción 4080871, Chile; ahaensgens@docente.uss.cl (A.H.); ligiai93@gmail.com (L.I.); bcontrerast@docente.uss.cl (B.C.-T.)

**Keywords:** microsynthesis, gold nanoparticles, AuNPs, biosensor, green nanotechnology

## Abstract

Gold nanoparticles (AuNPs) exhibit unique properties that make them appealing for applications in biosensing and other emerging fields. Despite the availability of numerous synthesis methods, important questions remain to be addressed regarding the volume effect on the synthesis yield and quality of AuNPs in the light of biosensing research. The present study addresses these issues by developing a novel microvolumetric citrate-reduction method to improve the synthesis of AuNPs, which were characterized by electronic microscopy, energy dispersive spectroscopy, zeta potential and colorimetric analysis. A comparison of the novel microsynthesis method with the standard Turkevich method demonstrated its superior performance in terms of yield, monodispersity, rapidity (in one step), reproducibility, and stability. The analytical behavior of AuNPs-based aptasensors prepared by microsynthesis was investigated using kanamycin detection and showed higher reproducibility and improved detection limits (3.4 times) compared to those of Turkevich AuNPs. Finally, the effect of pH was studied to demonstrate the suitability of the method for the screening of AuNP synthesis parameters that are of direct interest in biosensing research; the results showed an optimal pH range between 5.0 and 5.5. In summary, the approach described herein has the potential to improve research capabilities in biosensing, with the added benefits of lowering costs and minimizing waste generation in line with current trends in green nanotechnology.

## 1. Introduction

Gold nanoparticles (AuNPs) are of high interest in biosensing and a wide range of nanobiotechnology applications, due to a number of unique physical and chemical properties that are associated with their tunable size, shape-tailorable optical properties, and surface chemistry [1,2]. AuNPs-based biosensors based on surface plasmon resonance (SPR) have become thus a well-established approach for the label-free colorimetric detection of small molecules, providing a versatile, sensitive, selective, and easy-to-apply sensing platform [3]. The detection principle is typically based on the red-to-purple blue shift of the absorption spectrum during the aggregation of the nanoparticles [4].

A variety of synthesis methods have been described to obtain spherical AuNPs based on principles such as chemical reduction, solvothermal, electrochemical, photochemical, and sonochemical methods [2,5]. The Turkevich method is arguably the most widely employed protocol for the generation of citrate-stabilized spherical particles of 10 to 20 nm in diameter [6,7,8,9]. In this approach, the citrate salt acts as a reducing agent by forming a repulsion layer of citrate ions over the AuNPs’ surface that stabilizes growth, prevents aggregation, and keeps the particles in dispersion [10]. This method allows for the generation of stable monodisperse particles with narrow size distributions and good reproducibility [2,11,12], which are key conditions in conducting research on biosensing and a variety of emergent areas.

The relationship between the reducing agent and nanoparticle yield has been previously investigated in terms of the role of the components and the optimization of concentrations [13]. However, nanoparticle yield is an often-neglected issue in the discussion of nanoparticle synthesis, even though this parameter should, arguably, be considered from economic and practical application perspectives [14]. Likewise, the effect of the reaction volume on the performance and reproducibility of AuNPs synthesis has not been previously addressed [11]. The Turkevich method requires high volumes and amounts of chemical reagents to perform the synthesis (100–500 mL of 0.25 mM HAuCl_4_ solution are routinely prepared for a synthesis reaction), which tends to be inefficient for laboratory purposes typically associated with microvolume level assays and could become a potential source of AuNP wastes [15]. In addition, the use of *aqua regia* (a highly corrosive acid mixture) for glassware cleaning can potentially lead to hazardous waste generation [2,16].

The principles of green chemistry have become a reference guide for designing AuNPs synthesis routes with reduced use and production of hazardous chemicals, and which aim to be safer, simpler, cost-effective and environmentally friendly [17]. Green nanotechnology is thus a promising field that makes use of green chemistry concepts to improve the sustainability of nanotechnology [18]. An extensive research effort has been directed toward producing AuNPs through biosynthesis approaches based on plants, algae, yeast, fungi, and bacteria [5,19,20]. However, despite progress in these areas, the methodologies share several limitations, such as long synthesis times, raw material availability, the need for further processing, high heterogeneity and batch variability, and low production yields [17,21].

To the best of our knowledge, this is the first time a synthesis strategy has been proposed to address laboratory needs for the rapid availability of high-yield and high-quality AuNPs in a miniaturized assay format. This methodology produces AuNPs with excellent monodispersity, reproducibility, stability, and simplicity, thus offering the potential to accelerate the research and development of AuNPs biosensors and other AuNP-based applications. Finally, the microvolume scale significantly lowers the costs and minimizes waste generation, in line with current trends in green nanotechnology. 

## 2. Materials and Methods

### 2.1. Materials and Chemicals

Tetrachloroauric (III) acid trihydrate (HAuCl_4_ · 3H_2_O; 99.9%), trisodium citrate dehydrate, sodium chloride, hydrochloric acid, nitric acid, and kanamycin were purchased from Merck (Darmstadt, Germany). The single-stranded DNA aptamer was synthesized by Integrated DNA Technologies Inc. (IDT, Coralville, IA, USA). Commercially available AuNPs (Aldrich, Atlanta, GA, USA) were used as reference materials for spectroscopy, size, and morphology characterizations. All the solutions were prepared with ultrapure water (18.2 MΩ) obtained from a Millipore Simplicity Water System (Merck, Milford, MA, USA).

### 2.2. Turkevich Synthesis of Gold Nanoparticles (t-AuNPs)

An amount of 100 mL of the HAuCl_4_ solution (1 mM; pH 3.0–3.5) was boiled in a round-bottomed flask connected to a reflux and stirring system for 8 min. After that time, 10 mL of preheated solution of trisodium citrate 38.8 mM (pH 9.0) for 5 min at 60 °C were added. The mixture was kept boiling for 30 min until a reddish solution was formed, which was subsequently cooled down for 2 h at room temperature (without stirring) and filtered with a Millipore nylon filter (0.45 µm). Finally, the AuNPs solution was stored at 4 °C in the dark [22,23]. All glassware was washed with *aqua regia* and rinsed with ultrapure water obtained as indicated above. The resulting AuNPs will be referred to from now on as t-AuNPs.

### 2.3. Microsynthesis of Gold Nanoparticles (m-AuNPs)

An amount of 1 mL of the HAuCl_4_ solution (1 mM) was mixed with 0.1 mL of trisodium citrate 38.8 mM (volume ratio of 10:1 HAuCl_4_/citrate), which were prepared with ultrapure water. The final synthesis solutions were incubated in a block heater at 100 °C for 30 min. Then, the AuNPs solutions were cooled down to room temperature for 20 min and filtered with micropipette filter tips (polyethylene, 4 µm). The resulting AuNPs will be referred to from now on as m-AuNPs.

### 2.4. UV-Vis Spectroscopy

The concentration of AuNPs was determined spectrophotometrically with an Epoch^TM^ Microplate Spectrophotometer (Biotek Instruments, Winooski, VT, USA). 100 μL of the AuNPs solutions (optical pathlength 0.3 cm) were poured in triplicate into a 96-well microplate and the absorption spectra were recorded between 400–700 nm. The extinction coefficient (ε) was determined according to Liu et al. (2007) [24].

### 2.5. Dynamic Light Scattering (DLS) and Zeta Potential

The particle sizes and stability of the AuNPs were determined by DLS and zeta potential using a zeta-potentiometer (Zetasizer Nano-ZS90, Malvern Instruments, Westborough, MA, USA) at room temperature and a scattering angle of 90°. The Malvern Zetasizer Software version 7.12 was employed to analyze the collected data.

### 2.6. Electronic Microscopy

The core particle size and morphology of the AuNPs were determined by transmission electron microscope (TEM) with 4 Å resolution (JEOL-JEM 1200EX-II, Tokyo, Japan) and a Gatan CCD camera for image acquisition (model 782; Gatan, Inc., Pleasanton, CA, USA). Frequency histograms and morphology characterization (spherical index; SI) were determined by processing the TEM images with ImageJ software version 1.8.0_201 (Fiji) [25], where a value of 1 corresponds to a perfect sphere. Finally, elemental analysis was carried out using energy-dispersive X-ray spectroscopy (EDS) in a scanning electron microscope (SEM) with a resolution of 133 eV (SEM; JEOL-JSM 6380LV, Tokyo, Japan).

### 2.7. Nanoaptasensor Preparation 

AuNPs generated by both methods (m-AuNPs and t-AuNPs) were functionalized with a thiol-modified aptamer specific for kanamycin (5′C_3_-S-S-TGGGGGTTGAGGCTAAGCCGA-3′) [26], using the protocol described recently by Díaz-García et al. (2022) [27]. Briefly, the thiolated aptamer was reduced by incubation with dithiothreitol 0.1 M for 3 h in phosphate buffer (10 mM; pH 8) and subsequently purified by gel filtration with Sephadex G-25. Finally, The NAS was generated by incubating the AuNPs with aptamers (1:60 molar ratio), while stirring the mixture at 1200 rpm for 2 days in the dark (20 °C). 

### 2.8. Kanamycin Detection Assay

The resulting NAS were activated by heat at 80 °C for 10 min and then cooled down at room temperature for 10 min to induce linear conformation on the nanoparticle surface. Then, 200 µL of the antibiotic solution was incubated with 100 µL of the activated NAS (AuNPs 4 nM) at 60 °C for 10 min. and cooled down at room temperature. Finally, 60 µL of NaCl 1 M was added into the solutions and incubated for 30 min to monitor the aggregation process in terms of the shift of the SPR peak from 520 nm to 620 nm (A_520_/A_620_).

### 2.9. Screening of Synthesis Parameters: Effect of pH

The effect of pH on the absorption kinetics was investigated at four AuNPs synthesis conditions (pH 5.0, 5.5, 6.0, 7.0), by adjusting the pH with NaOH 1 M (1.1 mL final volume). The solutions were then incubated in a block heater at 95 °C during the evaluated time period (5, 10, 15, 20, 25 and 30 min) and the reactions were stopped by transferring the tubes to an ice bath for 30 min. The solutions were finally filtered with micropipette filter tips, stored in the dark at 4 °C and changes in the shape, intensity, and maximum wavelength of the SPR band were monitored with an Epoch^TM^ Microplate Spectrophotometer (Biotek Instruments, Winooski, VT, USA).

### 2.10. Statistical Analysis

Data are presented as mean ± standard error of at least three independent experiments. Statistical significance was determined at a 95% confidence level, using the non-parametric Mann–Whitney U-test to compare differences between two groups. Histograms for particle size and morphology characterization (spherical index) were obtained by measuring the diameter of at least 100 particles. The statistical significance of the slopes was calculated by Pearson correlation (*p* < 0.05) using linear regression analysis. The coefficient of variation (%CV) was employed to assess data reproducibility [28,29].

## 3. Results and Discussion

### 3.1. Spectroscopic and Physicochemical Characterization of AuNPs

**Figure 1:**
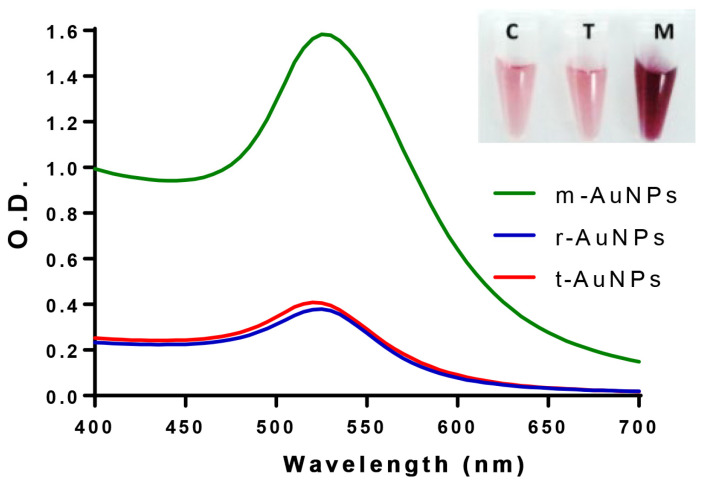
Spectroscopic characterization of m-AuNPs (green line), t-AuNPs (red line) and reference material (blue line). The inset image shows microtubes containing 100 mL of each AuNPs solution. The curves were averaged from three independent experiments (n = 3).

The absorption spectra of m-AuNPs and t-AuNPs were characterized and compared against reference material (r-AuNPs), showing the typical curves with a peak at 520 nm associated with the SPR band (Figure 1) [6,12]. Both t-AuNPs and r-AuNPs exhibited similar absorption spectra, with a maximum absorption intensity of 0.4 (at 520 nm). However, the solution of m-AuNPs achieved a 3.9 times higher absorption rate, accounting for a significantly higher concentration of nanoparticles (redder solution). 

**Figure 2:**
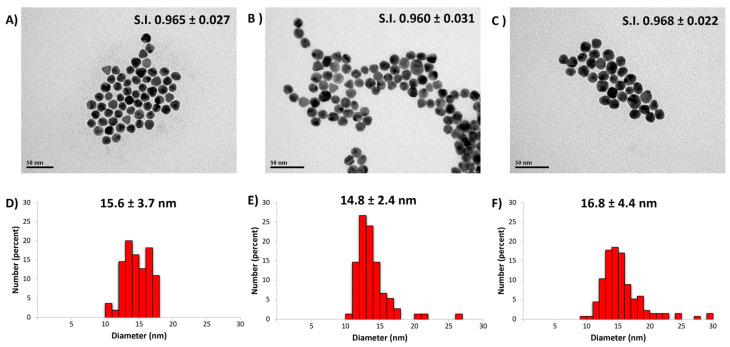
Size and morphology characterization of AuNPs: representative TEM images of r-AuNPs (**A**); m-AuNPs (**B**); and t-AuNPs (**C**). Histograms showing the particle size distributions obtained by TEM images for r-AuNPs (**D**); m-AuNPs (**E**) and t-AuNPs (**F**).

The well-defined and narrow shape of the SPR peak corresponding to m-AuNPs is consistent with a high nanoparticle sphericity. Indeed, a loss in the spherical shape would lead to an imbalance in the charge distribution during optical excitation of surface plasmons and the concomitant widening of the spectrum [30]. This spectroscopic behavior is further supported by the size and morphology distribution of the nanoparticles, as determined by TEM analysis (Figure 2). The three types of nanoparticles showed a high sphericity index (0.960 to 0.968), with average diameters of 15.6 nm for r-AuNPs (Figure 2A), 14.8 nm for m-AuNPs (Figure 2B), and 16.8 nm for t-AuNPs (Figure 2C), as well as a low dispersity in size (%CVs of 23.7, 16.2 and 26.2, respectively). Importantly, the m-AuNPs have a slightly lower diameter value (*p*-value of 0.001) along with a higher monodispersity (%CV 16.2). The excellent sphericity is a desirable condition for biosensing applications, as this shape provides useful attributes such as size- and shape-related optoelectronic properties, large surface-to-volume ratio, excellent biocompatibility, and low toxicity [6,31,32].

The terminology “high-yield” has been often employed in the field of AuNPs synthesis to account for processes where the resulting nanoparticles are highly uniform and homogeneous in terms of shape and size [33,34,35,36,37]. Despite a few studies actually performing accurate yield calculations with sophisticated equipment [37], simpler approaches have been proposed to shed light onto the efficiency of conversion between gold atoms in HAuCl_4_ and the nanoparticles. For example, the qualitative relationship between the concentration of gold salt and the intensity of the maximum absorption band has been applied to compare different results [14]. However, this analysis underestimates the differences in gold mass associated with variations in nanoparticle size. With this in mind, we developed an alternative analysis to estimate the number of gold atoms (Au^0^) that were converted into nanoparticle in relation to the number of gold atoms present in the synthesis solution (Au^3+^), as expressed in Equation (1). The calculation takes advantage of a previously reported correlation between the core diameter of spherical citrate-capped AuNPs with: (i) the molar extinction coefficient (Equation (2)); and (ii) the average number of gold atoms per AuNPs (Equation (3)) [24]. Based on this, we were able to determine gold conversion to AuNPs by first calculating the AuNPs molar concentration through the Beer–Lambert law (Equation (4)) to obtain the amount of gold atoms per AuNPs [24,38]:

(1) $$Y=\frac{\left[AuNPs\right]\cdot N_{a}\cdot A}{\left[Au^{3+}\right]\;\cdot N_{a}}$$

where Y is the synthesis yield of a given method, [AuNPs] is the concentration of gold nanoparticles (M), [*Au*^3+^] is the concentration of *Au*^+3^ (M) in the synthesis solution, *N_a_* is the Avogadro’s number (6.02214076 × 10^23^ moles^−1^) and *A* is the number of gold atoms per nanoparticle.

Ln ε = 3.32111 Ln D + 10.80505 (2)

where ε is the molar extinction coefficient (M^−1^cm^−1^) and D is the core diameter of AuNPs (nm).

(3) $$N=\frac{\pi \;\rho \;D^{3}}{6\;M}$$

where *N* is the number of gold atoms per AuNPs, ρ is the density of gold (19.3 g/cm^3^), *D* is the core diameter of AuNPs (nm), and *M* is the atomic weight of gold (197 g/mol).

*A* = ε c b (4)

where *A* is the absorbance at the SPR peak, c is concentration of AuNPs (M), ε is the molar extinction coefficient (M^−1^cm^−1^) and b is length of light path in centimeters (cm). 

Finally, the resulting yield was divided by the yield associated with the Turkevich method, with the aim of normalizing the method with respect to the most widespread and referential synthesis procedure.

(5) $$Y_{n}=\frac{Y}{Y_{t}}$$

where $Y_{n}$ is the normalized yield, Y is the yield of the method (e.g., microsynthesis) and $Y_{t}$ is the yield of the Turkevich method.

Table 1 summarizes the parameters determined for the nanoparticles, showing, for the m-AuNPs, a concentration (13.739 nM) several times higher than that obtained for the t-AuNPs (2.365 nM). This increase was 4 times greater, in terms of normalized yield ($Y_{n}$), with a concomitant reduction in the synthesis volume of ~100 times (see Section 2.2 and Section 2.3). It is worth noting that the yield increase is similar to that observed in the absorption of the SPR peak (Figure 1). 

Even though the Turkevich method is the oldest and most popular approach for the production of AuNPs, few advances have been made to improve the conversion rate of gold ions into nanoparticles. However, a recent study assessed, in a systematic manner, the effect on the synthesis of several variables (initial pH, temperature, order of reagent additions and the gold:citrate molar rate), shedding light on the optimal conditions for achieving higher yields than the standard protocol [39]. The specific conditions found in that study were a pH of 3 (HAuCl_4_ solution), a reaction temperature of 95 °C, citrate added to the boiling gold solution, and a molar ratio of 1:5 (HAuCl_4_/Na_3_Cit). Interestingly, the normalized yield estimated from these results was 4.4 times that of the yield typical when using the Turkevich method, which is slightly superior to our results when using microsynthesis (Table 1). It is also of note, however, that the effect of synthesis volume was not investigated in that study.

Previous studies investigating the use of green capping agents also provide some insight into the problem of AuNPs yield. For example, Jia et al. (2012) reported a high-yielding strategy based on a nonionic biosurfactant (ethoxylated sterol) [14], while Wang et al. (2009) studied the use of a chitosan–ninhydrin bioconjugate as a reducing and stabilizing agent [42]. By applying our calculation strategy, it was possible to compare these studies in a quantitative manner, showing increases of 1.8 and 2.75 times (Table 1). Therefore, microsynthesis achieved the highest performance in terms of AuNPs yield, providing high-quality features in terms of morphology, monodispersity, reproducibility, and stability.

The AuNPs obtained by microsynthesis and the Turkevich method were additionally characterized by zeta potential analysis (Figure S1). Table 2 summarizes the main parameters characterizing m-AuNPs and t-AuNPs, including the extinction coefficient (ε), the concentration of nanoparticles, the sphericity index, and zeta potential (pZeta). It is noteworthy that microsynthesis produced AuNPs with a much more negative pZeta value in comparison with AuNPs generated by the Turkevich method. This result suggests that the surface of the m-AuNPs possess higher levels of negative charges associated with citrate capping in comparison with the t-AuNPs, potentially improving the stabilization of the nanoparticles.

The chemical composition of the citrate-capped AuNPs is presented in Table 3, showing the relation between atomic gold and other elements in the sample (Ratio Au). The m-AuNPs show a higher amount of carbon per atom of gold in comparison with the t-AuNPs, which is likely associated with a higher amount of citrate in the stabilization layer. This result is consistent with the highly negative value of pZeta that was measured for the m-AuNPs (−35.1 ± 11.7 mV) in comparison with the t-AuNPs (−14.5 ± 10.9 mV). On the other hand, the m-AuNPs showed a lower amount of oxygen atoms (22.27 times higher than Au) in comparison with the t-AuNPs (85.3 times higher than Au). The increase in the carbon/Au ratio and the decrease in the oxygen/Au ratio are likely related to the lower amount of oxidized gold in the AuNPs.

The standard Turkevich method requires citrate to be added into a boiling HAuCl_4_ solution (typically refluxed in a round-bottomed flask), which might potentially alter the temperature uniformity. Indeed, the growth mechanism of the nanoparticles requires heating to overcome the energy barrier required for Au^0^ nuclei to grow by consuming the metal atoms in the bulk solution (coalescence). However, a slight reduction in the temperature (<90 °C) may cause an effect on the overall reduction rate (and the nucleation rate), leading to a decrease in particle size due to fewer seed particles initially produced compared to that achieved at higher temperatures [11]. The results presented herein show that a relevant increase in the synthesis yield of AuNPs can be achieved during a fast synthesis process in microvolume citrate-containing solution (“one-step synthesis”). 

According to the Fourier’s law of thermal conduction, the area:volume (A/V) ratio is a key parameter that controls the heat transference and affects the rate at which heat can be introduced into a reaction. This ratio decreases as the volume increases, causing the occurrence of inter-batch variations between synthesis reactions and preventing precise control over the size distribution of AuNPs [44]. This problem becomes a major issue for scaling-up purposes, as minimal variations in temperature might significantly alter the final product [44]. By contrast, the miniaturization of the volume conducted in this study has the potential to increase the rate of heat transference (higher A/V ratio) and, as a consequence, to contribute to the yield of the synthesis and improve the capping step and stabilization of the AuNPs. 

### 3.2. Effect of Volume on Reproducibility

**Figure 3:**
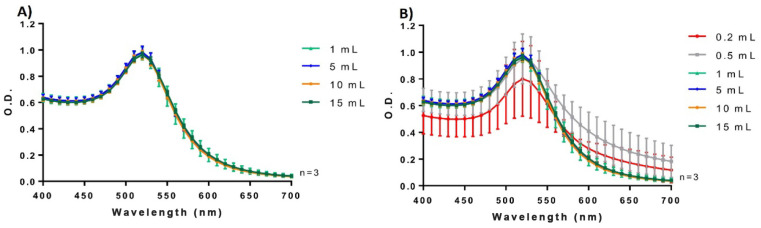
Spectroscopy characterization of microsynthesized AuNPs at pH 5.3 and different volumes (without pre-heat treatment): (**A**) absorption spectra of AuNPs obtained in 1, 5, 10 and 15 mL of synthesis solutions; and (**B**) absorption spectra of AuNPs obtained in 0.2, 0.5, 1, 5, 10 and 15 mL of synthesis solutions. Each curve represents the average from three independent experiments (n = 3).

To gain further insight into the volume effect, we carried out AuNPs microsynthesis in solutions containing volumes between 0.2 and 15 mL (at pH 5.3) and compared the results with, and without, a preheating stage for the HauCl_4_ solution (Figure 3 and Figure S2). The microsynthesis method performed excellently, with strong reproducibility, even without pre-heating of the HauCl_4_ solutions, with synthesis volumes between 1 and 15 mL (Figure 3A). Under these conditions the average fluctuations of the m-AuNPs spectra between repetitions was 4.06% (1mL), showing a similar spectroscopic behavior. By contrast, m-AuNPs synthesized in 0.2 mL exhibited a significant rise in absorbance beyond 550 nm, followed by a reduction in the SPR absorbance intensity indicative of a decrease in the monodispersity of the m-AuNPs (Figure 3B). An increase in the absorbances over 550 nm was observed for a volume of 0.5 mL, along with a higher O.D. variation in the spectra. By contrast, similar absorbance intensities were observed between 1 and 15 mL of the synthesis solution, suggesting that lower volumes are more susceptible to losing reproducibility in AuNPs synthesis. Interestingly, preheating reduced the batch variations from 9.4 ± 1.5% to 7.3 ± 1.5 (Table S1). Overall, these results lend support to the employment of 1 mL as the minimal synthesis volume.

### 3.3. Comparative Study of Stability

**Figure 4:**
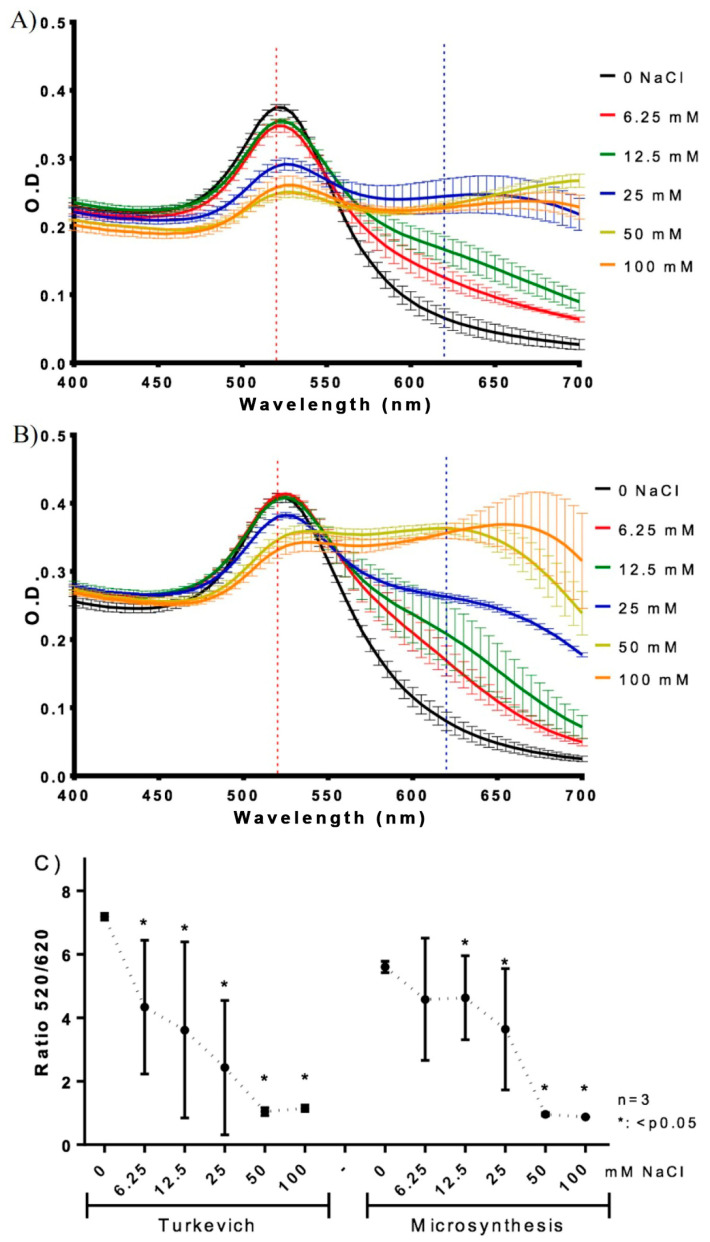
Salt-induced aggregation of: t-AuNPs: (**A**) and m-AuNPs (**B**) upon incubation with NaCl 0 to 100 mM. (**C**) Absorbance ratio 520/620 nm as a function of the concentration of NaCl. Results were averaged from three independent experiments (n = 3). Asterisks denote statistically significant differences between the treatments and controls. * = *p* < 0.05.

The aggregation of AuNPs obtained by microsynthesis and the Turkevich method was investigated by salt-induced aggregation, using fresh AuNPs and AuNPs that were stored for 3 years (aged AuNPs). The shift in the SPR peak from 520 nm (non-aggregated AuNPs) to 620 nm (aggregated AuNPs) is the preferred method for studying AuNPs aggregation through the decrease in the ratio 520/620 nm [27,45]. Figure 4 shows the spectrograms obtained in the presence of NaCl solution (0 to 100 mM) for t-AuNPs (Figure 4A) and m-AuNPs (Figure 4B), shedding light on their aggregation capacity and, therefore, the AuNPs’ stability. In both cases, a similar red-purple shift can be observed in the spectra. However, Figure 4C demonstrates that the m-AuNPs were less affected by salt-induced aggregation in comparison with the t-AuNPs, showing a significant decrease in the 520/620 ratio at NaCl concentrations of 12.5 mM (m-AuNPs) and 6.25 mM (t-AuNPs). This result complements previous findings in the EDS analysis and Zeta potential, providing further evidence about the increased level of citrate capping on the AuNPs produced by microsynthesis (Figure S1; Table 2 and Table 3), as the nanoparticles became less sensitive to aggregation. 

AuNPs solutions synthesized in the year 2020 via microsynthesis and the Turkevich method (stored at 4 °C) were characterized spectrophotometrically to gain insight into the long-term stability of both types of nanoparticles. To our knowledge this is a novel finding, given the long span of time covered by the present analysis (three years).

**Figure 5:**
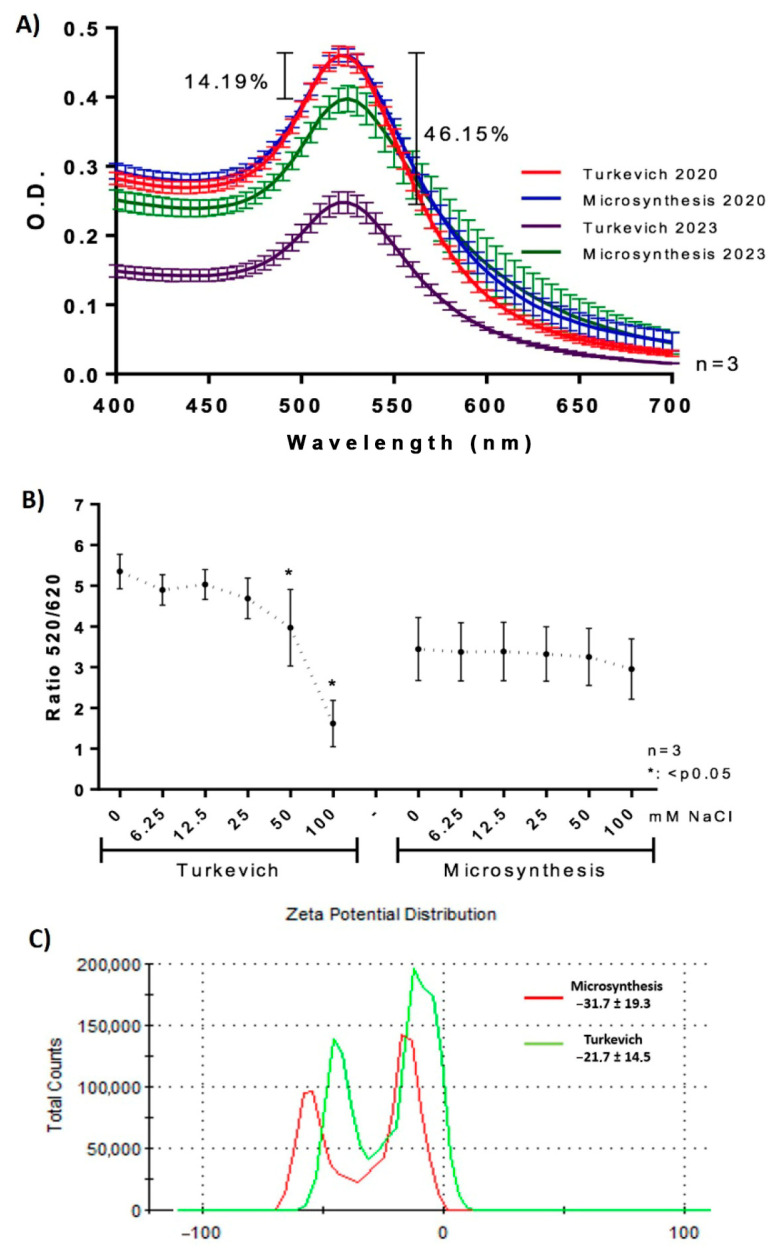
Long-term stability of AuNPs: (**A**) spectroscopic characterization of t-AuNPs and m-AuNPs after three years of storage; (**B**) absorbance ratio 520/620 nm as a function of the concentration of NaCl; and (**C**) zeta potential distribution (mV) of citrate-capped m-AuNPs (blue) and t-AuNPs (red). The results were averaged from three independent experiments for each AuNPs (n = 3). Non-significant differences are shown as n.s.

Figure 5A presents the absorption spectra of the AuNPs solutions generated in 2020 (red and blue) and 2023 (purple and green). Both spectra denote a decrease in the SPR band intensity, accounting for a loss of AuNPs in suspension (Figure S3). However, the decay associated with m-AuNPs (14.19%) is much less than that of the t-AuNPs (46.15%). Figure 5B presents the salt-induced aggregation of both types of AuNPs, which were incubated with 100, 50, 25, 12.5, 6.25 and 0 mM of NaCl.

No significant decrease in the 520/620 nm absorption ratio was observed between 3-year stored AuNPs obtained by the Turkevich and microsynthesis methods. However, while t-AuNPs presented a significant drop in the ratio at NaCl concentrations of 50 nM and 100 mM, the m-AuNPs’ curve denotes a rather constant behavior in this parameter across all NaCl concentrations. As expected, aged m-AuNPs showed a decrease in salt-induced aggregation in comparison with fresh m-AuNPs. These results confirm the higher stability of m-AuNPs, even after three years of storage.

The surface charge of m-AuNPs (25.7 mV ± 18.3) was higher than that of t-AuNPs (31.7 ± 19.3 mV), according to zeta potential analyses (Figure 5C). Previous works have shown that, over the course of AuNPs’ aging, gold atoms move to the top of the nanoparticles and bind on the surface to terminal carboxylates of the citrate molecule [46]. Accordingly, a high citrate coverage would increase the stability of AuNPs by preventing the loss of gold from their core and potentially becoming less susceptible to the loss of gold atoms during long-term storage. This effect is consistent with the appearance of a black precipitate in Falcon tubes containing aged AuNPs (15 mL) and the concomitant decrease in the gold concentration in the suspension (Figure S3). 

It is of note that currently there is a growing interest in developing new green synthesis approaches to synthetize AuNPs by replacing chemical reducing agents with plant extracts or bioactive compounds [20], which potentially provide stronger capabilities to reduce metal ions into metal nanoparticles [47,48]. Biogenic approaches have been discussed as promising methods for AuNPs synthesis because of the utilization of cost-effective and non-hazardous raw materials, the ease of synthesis, and safety aspects [49,50]. However, despite these advantages being well-grounded in the light of current trends of green nanotechnology, these methods still face challenges related with the heterogeneity of the green materials, their inherent variability, the presence of impurities that often result in nanoparticles with less uniform sizes, and limited control over surface modification [2,5,19,51,52].

Finally, the costs of both methodologies were compared by standardizing the needs of materials and reagents for equal volumes (100 mL; Table S2), which showed that microsynthesis was significantly cheaper (70.77 USD) compared to the standard Turkevich method (145.45 USD). In addition, the synthesis of m-AuNPs was 16 times faster than that of t-AuNPs (1.5 h in comparison with 24 h). Therefore, microsynthesis largely outperformed the Turkevich method in terms of rapidity and cost-effectiveness. Another difference between the approaches is associated with the requirement of *aqua regia* for treating glass materials (Turkevich method), which has an impact on both the cost and the environmental effects associated with these residues. Synthesis optimization plays a pivotal role in achieving both high-yield and cost-effective production of tailored-size AuNPs that meets green nanotechnology principles, which are desirable conditions to take advantage of when using AuNPs in biosensing and other bioapplications [39]. Thus, our microvolume-scale approach enabled a high-performing yield of AuNPs, while simplifying the procedure, reducing the costs, and minimizing the generation of hazardous wastes, which is strongly in line with current trends in green synthesis and green nanotechnology.

### 3.4. Antibiotic Detection Assays with Nano-Aptasensors (NAS)

AuNPs are of paramount importance in biosensor development thanks to their optical properties, which enable the label-free colorimetric detection of a wide range of analytes [1,53,54]. In this context, the capability of aptamers to recognize a wide range of analytes makes AuNPs-based NAS a versatile platform for sensing applications, with a fast response, and high sensitivity, selectivity, and reliability [55,56,57].

**Figure 6:**
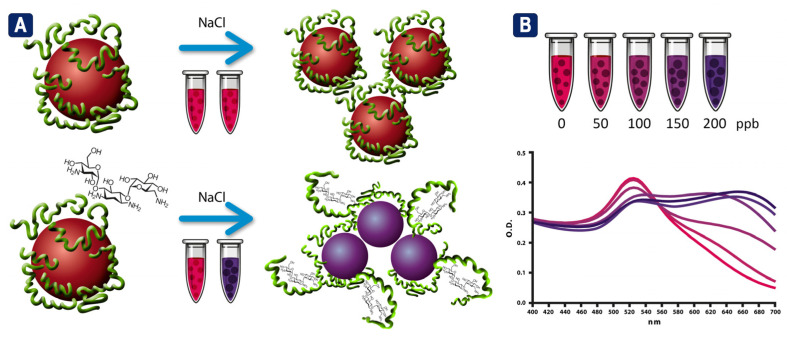
Schematic illustration showing the sensing principle of aptamer-conjugated AuNPs for colorimetric detection of kanamycin: (**A**) covalently conjugated aptamers inhibit salt-induced aggregation, while conformational change induced by interaction with the antibiotic decreases surface protection and leads to AuNPs aggregation upon NaCl addition; and (**B**) absorption spectra of AuNPs showing the shift in the plasmon resonance peak (from red to blue purple) in response to antibiotic-induced aggregation.

AuNPs-based NAS were developed and assessed to investigate the use of AuNPs generated by both methods in the detection assays of kanamycin (0–200 ppb). Figure 6 provides a schematic description of the detection reaction, showing aptamer-coated AuNPs stabilized by electrostatic repulsions. In the presence of kanamycin, aptamers adopt a folded structure that leads to a decrease in surface protection and the subsequent aggregation of the AuNPs upon the addition of NaCl. The aggregation process is proportional to the antibiotic concentration and can be followed through the decrease in the absorption ratio between 520 nm and 620 nm (A_520_/A_620_).

**Figure 7:**
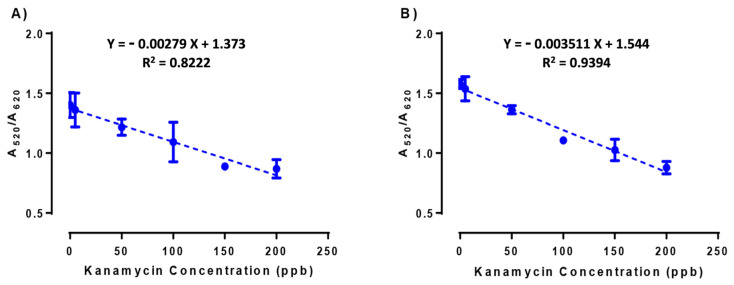
Kanamycin detection assays with AuNPs-based NAS obtained by (**A**) Turkevich and (**B**) microsynthesis methods. Spectroscopic data for each condition were determined in triplicates (n = 3).

AuNPs aggregation data were analyzed spectrophotometrically by measuring the shift of the plasmon resonance peak from 520 nm to 620 nm (A_520_/A_620_). Despite both methods showing a similar linear behavior, NAS based on m-AuNPs delivered a higher coefficient of determination (r^2^ = 0.9475) and less dispersion among the repetitions (Figure 7). 

It is noteworthy that the LOD value determined in this study (19.15 ppb) is lower than the value obtained in a recent study reported by our group on the detection of kanamycin (37.5 ppb) using the same NAS prepared, in that case, using the Turkevich method [27]. Table 4 summarizes the analytical parameters accounting for the performance of the two NAS, including the detection limit (LOD), quantification limits (LOQ), variation coefficient (%CV), and standard errors. The m-AuNPs-based NAS outperformed the t-AuNPs-based NAS in terms of the increase in sensitivity, by reducing the LOD (19.15 ppb) by 3.4 times, and the LOQ (61.45 ppb) by 3.5 times. This difference is a direct consequence of the higher reproducibility of the analytical data generated by m-AuNPs-based NAS (%CV > 2.4). 

A key parameter impacting on biosensor’s sensitivity, selectivity and stability is the homogeneity of the resulting AuNPs solution, as variations in particle size and shape may cause inconsistent surface areas and effects on the plasmonic properties [58]. These issues can result in non-uniform binding of ligands or receptors on the AuNPs surface, affecting the recognition of analytes, the response time, and the optimal performance of biosensors [59,60,61]. In this context, the high dispersity, reproducibility, and stability achieved by m-AuNPs make them excellent candidates for research and development of AuNPs-based biosensors.

### 3.5. Other Bioapplications: Screening of Synthesis Parameters

Microsynthesis provides an excellent tool to be applied in the screening of synthesis parameters to optimize the properties of nanoparticles for biosensing research and within emerging fields including vaccination [62], diagnostics [63], imaging [64] and therapies [65]. As an example, we have successfully applied AuNPs microsynthesis for conjugation and delivery of peptides (results to be published elsewhere).

The Turkevich method is a simple and reproducible technique. However, the process can be affected by several parameters such as pH fluctuations and citrate availability during synthesis [12], Au:citrate molar ratios [66], polarity of solvents used during the synthesis [67], the order of addition of the reagents [68], among several other conditions that are often investigated [11,31,69]. Importantly, the method reported herein is appropriate for straightforward and systematic study of conditions and reaction parameters. These experiments are otherwise difficult to perform with traditional Turkevich method due to the high amounts of materials/reagents, the high volumes (from 100 mL) and the complexity of the protocols.

**Figure 8:**
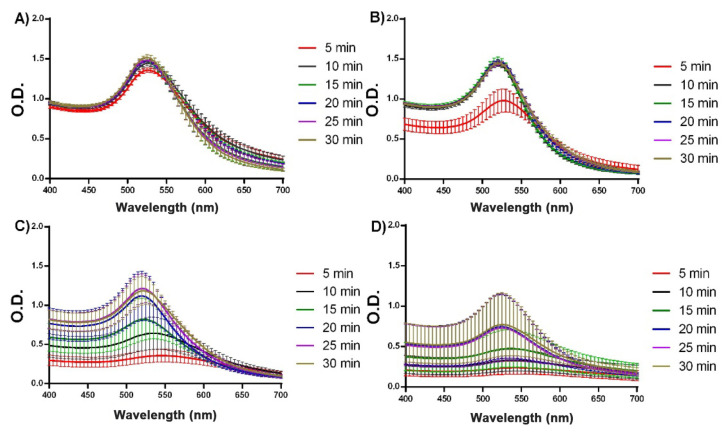
Spectroscopic characterization of the kinetics of AuNPs formation at times ranging between 5 and 30 min after synthesis (red, black, green, blue, purple and olive lines, respectively), showing the effect of the pH at values of 5.0 (**A**); 5.5 (**B**); 6.0 (**C**); and 7.0 (**D**).

The pH of the solution is known to be crucial in controlling the size, morphology, and stability of the AuNPs produced via the citrate reduction method [11]. Previously, we have demonstrated that slight pH fluctuations between 4.7 to 5.3 have an impact on the performance of the Turkevich method, affecting the yield in a pH-dependent manner [12]. To learn more about pH effects over the AuNPs synthesis, we employed microsynthesis to investigate its relationship with the kinetics of AuNPs formation. Figure 8 presents the behavior of the spectra (SPR band) in the time range assessed (5 to 30 min) at pH 5.0, 5.5, 6.0 and 7.0. As expected, as the reaction proceeds all the curves show an increase in the intensity of the SPR peak, which accounts for an enhancement of the number of particles produced along the time line. In addition, the absorption maxima shifted in all cases to the left (centering around of 520 nm), denoting an increase in the particle number accompanied by a reduction in particle size in time [70]. 

The differences in the spectra associated with different pH values were noticeable, proving that a pH of 5.0–5.5 was the best range in terms of keeping the narrow distribution of nanoparticle sizes (related with the width of the peaks) and minimizing the dispersion in the absorbance values among the repetitions. The highest reproducibility at a pH of 5.0 was obtained at 10 min of synthesis, with a negligible standard error (Figure 8A). At higher pH values, the observed trend is a reduction in the intensity of the SPR peak (i.e., lesser particle formation), with a simultaneous decay in the reproducibility of the resulting curves. At a pH of 5.5, the increase in the variability becomes relevant at 5 min of synthesis (Figure 8B), reaching the worst effect at a pH of 6.0 (Figure 8C) and 7.0 (Figure 8D). It is worth mentioning that the shift of the SPR peak to 540 nm at pH 7.0 (5 min) is consistent with a diameter of 60 nm [71]. 

These findings are in agreement with a previous study showing, at high pH values, an increase in the dispersity of the optical signals of AuNPs [72]. Likewise, the behavior of the spectra is consistent with the seed-mediated growth mechanism and confirms our previous finding of pH 5.3 as a preferred condition favoring the synthesis of AuNPs by improving citrate absorption onto the nanoparticle surface [27]. 

Gold species present in a solution undergo complex equilibria as a function of the pH, leading to the formation of various gold ions and hydroxide complexes. Citrate, likewise, plays a central role by reducing gold ions (Au^3+^) into gold atoms (Au^0^) and stabilizing the formed nanoparticles. Thus, Au^3+^ becomes the predominant species at a pH of 5.0 and 5.5, while at a pH of 6.0, a mixture of Au^3+^ and gold hydroxide ions (AuOH_2_^+^) exists. As the pH increases to 7.0, the concentration of Au^3+^ decreases and gold hydroxide (AuOH_3_) becomes dominant. Therefore, at a pH 5.0 and 5.5, the high concentration of Au^3+^ in the presence of deprotonated citrate strongly favors the reduction of Au^3+^ and improves the performance of the synthesis. However, as the pH increases to 6.0 and 7.0, the presence of gold hydroxide complexes competes with the availability of Au^3+^ and affects the rate of nanoparticle formation [11,72].

Therefore, the microvolume format allows for multiple conditions and parameters associated with the synthesis methodologies to be easily and rapidly investigated, including pH, citrate concentration, molar ratios, novel coating agents, temperature, and incubation times, making this method a valuable tool to accelerate the research and development of biosensing and other fields.

## 4. Conclusions

A novel “one-step” methodology to synthesize AuNPs in a microvolume citrate-containing solution was developed and thoroughly characterized, providing an easy and efficient means to perform research on biosensing. The microsynthesis method achieved a superior performance in terms of yield, rapidity, reproducibility, stability, and cost, enabling a cost-effective supply of high-quality and high-yield nanoparticles. The yield was similar to that of an optimized Turkevich protocol, but four times higher than that obtained using a standard Turkevich method; however, the microsynthesis requires only 1.1 mL of synthesis volume and costs approximately half as much. In addition to reducing the volume and requirements for gold and other chemical reagents, the microvolume-scale avoids the use of *aqua regia* (a highly corrosive acid mixture) and minimizes the generation of hazardous wastes. Therefore, it is a methodology that combines the high quality of citrate reduction (chemical) methods with current trends in green synthesis and green nanotechnology by virtue of its superior cost-effectiveness and its lower impact on the environment. Regarding biosensing applications, NAS prepared by microsynthesis demonstrated a superior analytical performance compared to that of the Turkevich method in terms of reproducibility, detection, and the quantification limits of kanamycin. Finally, the microvolume format provided a rapid and easy means to investigate multiple conditions and parameters associated with the synthesis methodology. The proposed experimental strategy addresses laboratory needs and holds the potential to accelerate research on biosensing and other bioapplications.

## Figures and Tables

**Table 1:** Comparative analysis of AuNPs synthesis yields in terms of gold atoms in the synthesis solution converted into gold atoms in AuNPs and normalized with respect to the Turkevich method.

Reference	Gold in Synthesis Solution (mM)	AuNPs Diameter (nm)	Extinction Coefficient (M^−1^ cm^−1^)	O.D. (1 cm)	AuNPs (nM)	Gold Atoms/AuNPs	Y	Y_n_
* This work (m-AuNPs)	1	14.8	379,438,572	5.21	14	100,158	137.5	4
* This work (t-AuNPs)	1	16.8	578,043,471	1.36	2.4	146,498	34.6	1
* Bahmanyar et al. 2022 [39]	0.29	15	396,736,386	1.67	4.21	104,274	151.4	4.4
** Jia et al. 2012 [14]	0.5	24.3	1,969,356,963	2.11	1.1	443,324	95.0	2.7
*** Gangula et al. 2011 [40]	0.6	25	2,164,143,895	0.56	0.26	482,750	20.8	0.6
Qiao et al. 2011 [41]	1	20	1,031,424,209	0.38	0.37	247,168	9.1	0.3
*** Wang et al. 2009 [42]	0.243	42	12,121,686,353	0.8	0.066	2,289,024	62.2	1.8
** Huang et al. 2009 [43]	0.5	11.5	164,160,171	0.7	4.26	46,989	40.1	0.29

* Synthesis using Turkevich-based methodology. ** Synthesis using biomolecules as capping/reduction agent. *** Synthesis using plant extracts as capping/reduction agent.

**Table 2:** Characterization parameters of AuNPs obtained by the Turkevich and microsynthesis methods.

Method	Diameter (nm)	Concentration (nM)	%CV	Sphericity Index	p Zeta (mV)	Synthesis Yield
Turkevich	16.8	2.365	26.2	0.97	−14.5 ± 10.9	1
Microsynthesis	14.8	13.739	16.2	0.96	−35.1 ± 11.7	4

**Table 3:** EDS elemental analysis of AuNPs obtained by the Turkevich and microsynthesis method.

	Turkevich Method		Microsynthesis Method	
Elements	Atomic %	Ratio Au	Atomic %	Ratio Au
Au M	0.98	1	3.07	1
C K	2.03	2.071	16.81	5.48
O K	83.59	85.30	68.36	22.27
Na K	13.40	13.67	11.76	3.83

**Table 4:** Comparison of analytical parameters of NAS based in m-AuNPs and t-AuNPs.

Method	Standard Error	%CV of Zeros	LOD (ppb)	LOQ (ppb)
Turkevich	0.07740	7.13	64.21	217.79
Microsynthesis	0.04564	2.37	19.15	61.45

## Data Availability

The data presented in this study are available on request from the corresponding author. The data are not publicly available due to privacy restrictions.

## Supplementary Materials

The following supporting information can be downloaded at: https://www.mdpi.com/article/10.3390/bios13120992/s1, Figure S1: Zeta potential distribution (mV) of citrate-capped m-AuNPs (blue), and t-AuNPs (red); Figure S2: Spectroscopy characterization of microsynthesized AuNPs at different volumes with (blue segmented Line) or without pre-heat treatments (red line). Figure S3: Image of falcon tubes that containing the m-AuNPs and t-AuNPs solutions for three years; Table S1: Spectroscopy variation of microsynthesized AuNPs at different volumes with or without pre-heat treatments; Table S2: Cost analysis and comparison between Turkevich and Microsynthesis methods.
