# Supplementary material for: Novel Microsynthesis of High-Yield Gold Nanoparticles to Accelerate Research in Biosensing and Other Bioapplications
Source: Biosensors (Basel). 2023 Nov 21;13(12):992. doi: 10.3390/bios13120992 (PMC10742281; doi:10.3390/bios13120992)
Supplement: Supplementary file 1 [file biosensors-13-00992-s001.zip › biosensors-2653713-supplementary.pdf]

Supplementary material to the manuscript

Novel microsynthesis of high-yield gold nanoparticles to accelerate research in biosensing and other bioapplications

Víctor Díaz-García*, Astrid Haensgen, Ligia Inostroza, Eduardo Zúñiga, Braulio Contreras-Trigo, Patricio Oyarzún*

Facultad de Ingeniería, Arquitectura y Diseño, Universidad San Sebastián, Lientur 1457, Concepción 4080871, Chile

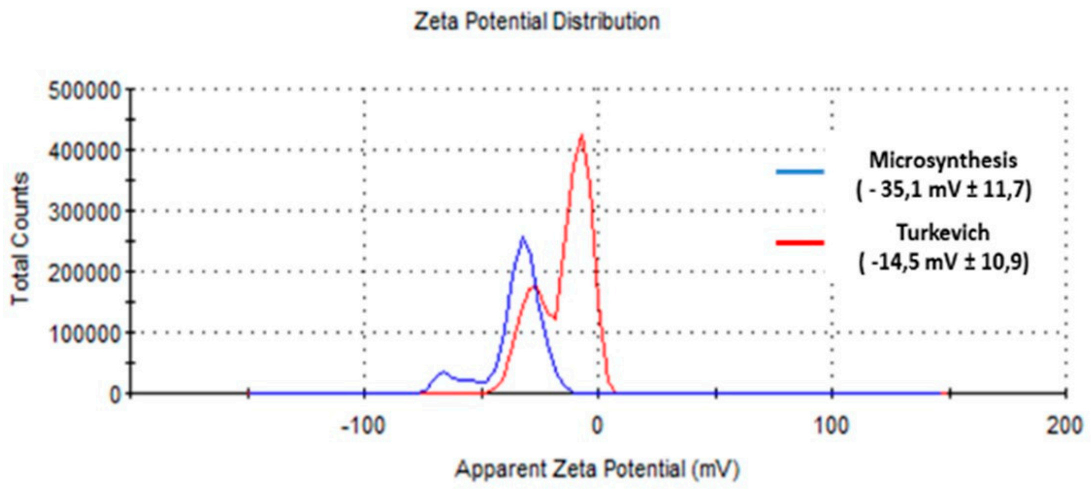

Figure S1: Zeta potential distribution (mV) of citrate-capped m-AuNPs (blue), and t-AuNPs (red). Inset shows the Zeta potential values determined for each sample.

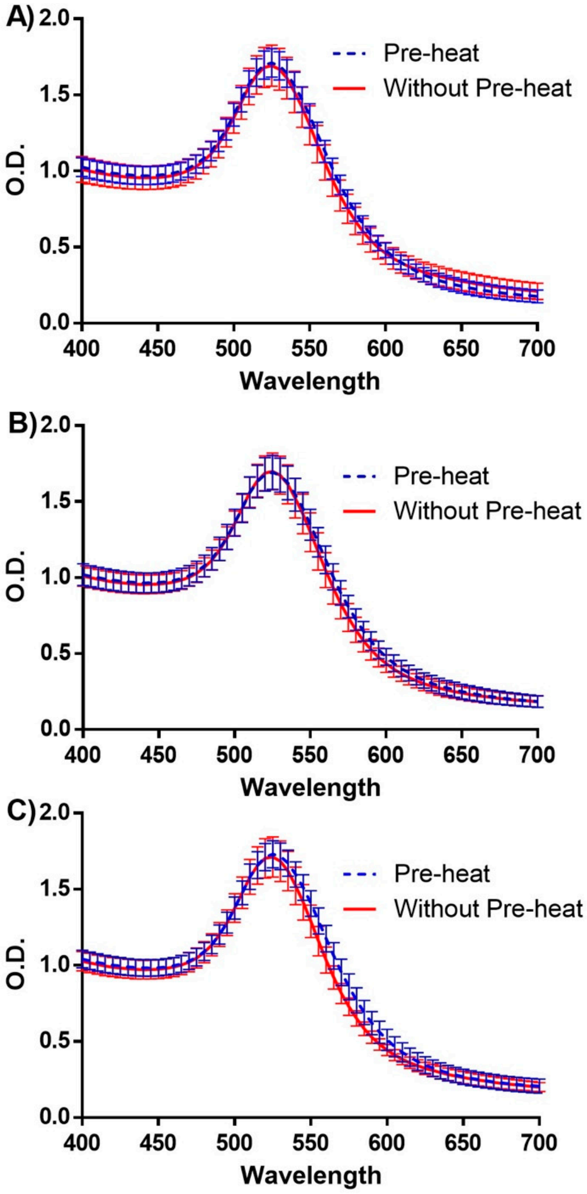

Figure S2: Spectroscopy characterization of microsynthesized AuNPs at different volumes with (blue segmented Line) or without pre-heat treatments (red line). A) Absorption spectra of AuNPs obtained in 5 mL of synthesis solutions. B) Absorption spectra of AuNPs obtained in 10 mL of synthesis solutions. C) Absorption spectra of AuNPs obtained in 15 mL of synthesis solutions. Each curve represents the average from 3 independent experiments (n = 3).

m-AuNPs t-AuNPs

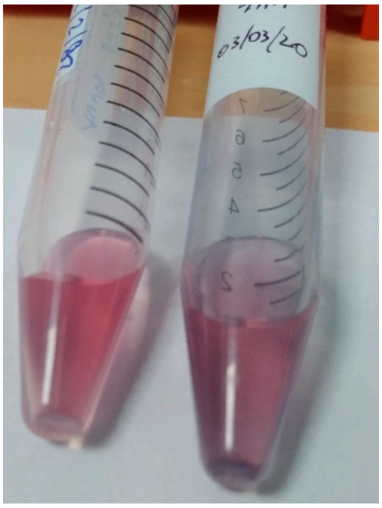

Figure S3: Image of falcon tubes that containing the m-AuNPs and t-AuNPs solutions for 3 years. Notes the black precipitate associated to gold precipitation.

Table S1: Spectroscopic variation of microsynthesized AuNPs at different reaction volumes with and without pre-heat treatments. Each value represents the average from 3 independent experiments (n = 3).

Area under the curve (400-700nm)	5mL		10 mL		15 mL	
	Pre-heat	without Pre-heat	Pre-heat	without Pre-heat	Pre-heat	without Pre-heat
Average	252,3	249,1	251,2	245,4	259,4	249,4
SD	17,7	24,7	19,7	20,6	17,0	16,9
%CV	7,0	9,9	7,9	8,4	6,6	6,8

Table S2: Costs analysis and comparison between Turkevich and Microsynthesis methods.

		Turkevich method		Microsynthesis method	
Materials	Price (US$)/Amount	Required amount	Cost (US$)	Required amount	Cost (US$)
HCl 37 %	42/2.5 L	150 mL	2,52	-	-
HNO3 69 %	57/2.5 L	50 mL	1,14	-	-
HAuCl4 99 %	516/1 g	40 mg	20,64	40 mg	20,64
Na3-Citrate	96/500 g	114 mg	0,02	114 mg	0,02
Nanopure water	43/4 L	3 L	32,25	110 mL	1,18
Nylon filter (0.45 µm)	72/200	1 unit	0,36	-	-
Micropipette Filter Tips	12/100 units	-	-	20 units	2,4
Microtube	25/1000 units	-	-	100 units	2,5
Drinking water	0.8/1000 L	600 L	0,48	-	-
Electricity	0.18/KWh	250 KWh	0,04	153 KWh	0,03
Human resource	22/h	4 h	88	2 h	44
Total Cost (US$)			145,45		70,77
